# Non traumatic acquired acute transdiaphragmatic intercostal hernia induced by coughing

**DOI:** 10.1186/s13019-023-02320-3

**Published:** 2023-07-04

**Authors:** June Lee, Ju Sang Kim, Jin Yong Jeong

**Affiliations:** 1 Department of Thoracic and Cardiovascular Surgery, Seoul St. Mary’s Hospital, College of Medicine, The Catholic University of Korea, Seoul, Republic of Korea; 2 Department of Internal Medicine, Incheon St. Mary’s Hospital, College of Medicine, The Catholic University of Korea, Seoul, Republic of Korea; 3 Department of Thoracic and Cardiovascular Surgery, Incheon St. Mary’s Hospital, College of Medicine, The Catholic University of Korea, 56 Dongsu-ro, Bupyeong-gu, Incheon, 21431 Republic of Korea

**Keywords:** Rupture, Thoracic wall, Abdominal wall, Diaphragm, Cough, Hernia

## Abstract

Transdiaphragmatic intercostal hernia is a rare disease. It is usually caused by trauma and is rarely caused by coughing. Although a few cases of intercostal hernia induced by coughing have been reported, our case of a non traumatic acquired acute transdiaphragmatic intercostal and abdominal hernia induced by coughing is very rare. A 77-year-old woman presented with sudden-onset left lower chest pain after an episode of violent coughing. She had risk factors for intercostal hernia, including obesity, chronic obstructive pulmonary disease, oral steroid use, and diabetes mellitus. Computed tomography showed herniation of the lung and intra-abdominal organs into the thoracic and abdominal wall through a ruptured diaphragm, as well as the intercostal and abdominal muscles. Surgery was completed with interrupted sutures to close the defects after the reduction of the herniated organs. Our experience suggests that careful examinations, including the assessment of risk factors and computed tomography imaging, were essential for establishing an accurate diagnosis, and that the repair of a ruptured diaphragm with simple interrupted sutures without any prosthetic materials seems to be feasible in selected patients with a transdiaphragmatic intercostal hernia.

## Correspondence

**Dear Sir**,

Recently, an unusual case of transdiaphragmatic intercostal hernia (TDIH) with delayed onset and history of blunt trauma incurred in a high-speed motor vehicle accident has been reported [1]. TDIH is a rare disease and usually develops after a traumatic event. However, spontaneous cough-induced TDIH is extremely uncommon. Recently, we encountered a rare case of an extensive TDIH with acute-onset, which was induced by coughing and was not associated with a trauma history. Herein, we describe successful repair of a cough-induced TDIH via thoracotomy focused on the feasibility of primary sutures in the acute phase.

**Fig. 1:**
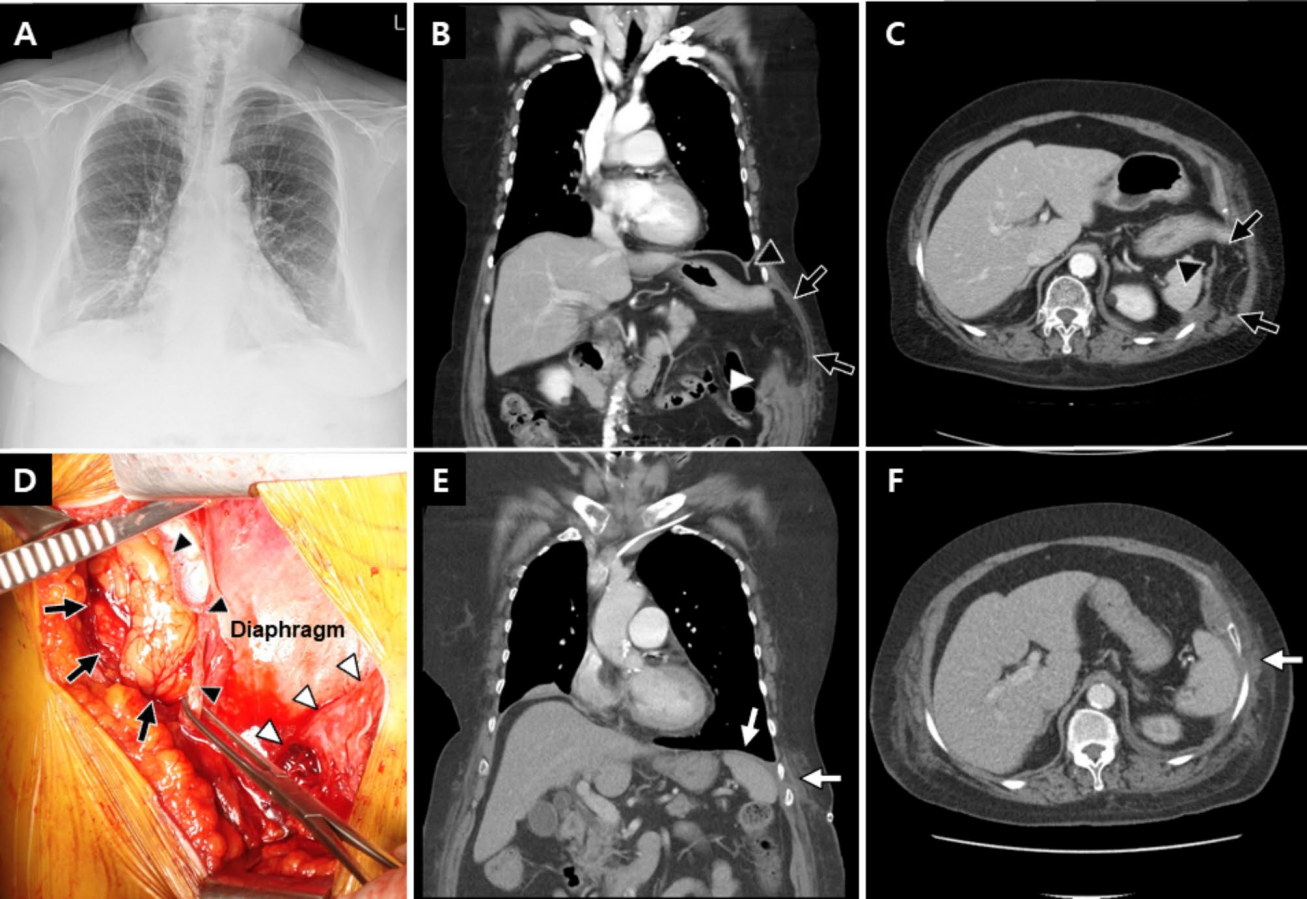
Chest X-ray and thoracoabdominal computed tomography (**A**, **B** and **C**) before surgery showing herniation of the lung and intra-abdominal organs into the thoracic and abdominal walls (black arrows) through the ruptured diaphragm (black arrowhead), as well as the intercostal and abdominal muscles (white arrowhead); the operative findings (**D**) demonstrating herniated intra-abdominal organs (black arrows) through the ruptured diaphragm (black arrowheads) and parietal pleura (white arrowheads); the thoracoabdominal computed tomography (**E**, **F**) after surgery revealing reduced herniation, an intact diaphragm, and repair of the left ninth intercostal space (white arrows)

A 77-year-old obese woman with a history of long-term steroid use for asthma and diabetes mellitus presented with sudden-onset left lower chest pain after an episode of violent coughing. She had no history of blunt trauma to the chest or abdomen. Physical examination revealed soft tissue bulging on the left lower chest wall. The chest wall exhibited ecchymosis. The laboratory results showed increased levels of white blood cells (11.80, 4.0–10.0 × 10^9^/L) and serum creatine phosphokinase (373, 0–250 IU/L). CT showed herniation of the basal portion of the lower lobe of the left lung and intra-abdominal organs, including the stomach, left colon, and mesenteric fat, into the thoracic and abdominal walls through the ruptured diaphragm, as well as the abdominal and ninth intercostal muscles (Fig. 1A, B and C). Chest wall hematoma and emphysema were also present. Surgery was urgently performed. After endotracheal general anesthesia, the patient was placed in the right down decubitus position. Through an anterolateral thoracoabdominal skin incision 20 cm in length, we confirmed a 20-cm herniated sac in the ninth intercostal space and about a 10-cm ruptured diaphragm defect on the anterolateral aspect (Fig. 1D). Reduction of the abdominal organs into the peritoneal cavity was carried out. The repairs were completed with one or two rows of interrupted sutures (2–0 coated braided polyester) to close the defects without any biologic mesh. We confirmed the absence of tension at the repair site and proceeded to close the wound layer by layer after inserting a chest tube. The chest tube was removed on the 7th postoperative day, and the patient was discharged on the 30th day after surgery following treatment for asthma, diabetes control, and wound management. The postoperative CT scan revealed a well-repaired diaphragm and intercostal space (Fig. 1E, F). There were no significant complications during hospitalization. After being referred by internal medicine, the patient continued to receive continuous treatment for asthma, diabetes, and obesity as an outpatient. The patient has been followed up for 5 years in our hospital without hernia recurrence.

TDIH is commonly caused by trauma involving motor vehicle accidents, and it may be diagnosed during admission to the emergency department [2]. Mohandas et al. reported a case of TDIH with delayed onset and history of blunt trauma history experienced in a motor vehicle accident [1]. Usually traumatic diaphragmatic hernia occurs more at left side than right like our case [3]. Desforges et al. [4] stated that the left hemidiaphragm has multiple structural weaknesses because of the presence of several points, such as the esophageal hiatus, aortic hiatus, and foramen of Morgagni. Furthermore, the liver on the right side provides extra support to the right hemidiaphragm, making it less susceptible to rupture. Rarely, TDIH can occur spontaneously, and symptoms may be delayed even in these circumstances [5]. However, in our case, the patient experienced spontaneous TDIH with symptoms that occurred suddenly after severe coughing.

Although a few cases of cough-induced intercostal hernia have been reported [5–7], our case of an extensive cough-induced transdiaphragmatic intercostal and abdominal hernia with lung and intra-abdominal organ herniation is very rare. Spontaneous TDIH may be difficult to diagnose based on patient symptoms alone because the disease entity is very rare and the herniated organs are not exposed outside the body. Maeda et al. described the following two useful findings for spontaneous intercostal hernia: painful chest wall ecchymosis and unexplainable elevation of non-cardiogenic creatine kinase [8]. The former entity requires imaging because it is likely to exclude spontaneous rib fracture, and ecchymosis is the third most common finding after bulging and pain. The latter finding is most likely associated with damage to the chest wall muscles related to hernias. Intercostal hernias occur due to risk factors, including obesity, chronic obstructive pulmonary disease, oral steroid use, and diabetes mellitus [6]. Our patient had all of these risk factors. Contrast-enhanced CT of the chest and abdomen is the gold standard for diagnosing diaphragmatic rupture with high sensitivity and specificity [5]. Radiologic findings including collar sign or using nasogastric tube are helpful for quick diagnosis [3].

There are several surgical options for the treatment of TDIH including primary closure, using mesh, and closure with prosthetic or autologous material [9]. In acute state of diaphragmatic hernia, primary repair of diaphragm is reasonable. In chronic diaphragmatic hernia or a large size of defect, repair using mesh could be useful for a tension free repair [3]. Unlike our case of acute phase, Daniel R et al. [10] reported a chronic diaphragmatic hernia through 8th intercostal space, complicated by rib fracture, which was repaired with primary closure. We tried primary closure first on the diaphragmatic and intercostal space defects and checked the tension on the sutures. After confirming the solidity of the repaired site, we decided to finish the operation. We would like to emphasize that in this case, we were able to diagnose acute diaphragmatic rupture rapidly and accurately, and perform direct repair successfully without biologic mesh. The patient maintained good condition during the long-term follow-up. Additionally, we highlight the importance of managing underlying conditions such as diabetes, pulmonary diseases requiring steroid use, and obesity under the supervision of internal medicine to ensure long-term maintenance.

In summary, we encountered a rare case of an extensive transdiaphragmatic intercostal and abdominal hernia without a trauma history to the thoracoabdominal wall, with the sudden onset of symptoms after an episode of violent coughing. Through our experience, we would like to emphasize that careful examinations, including suspicion based on symptom history, the assessment of risk factors, physical examination, and CT imaging, are essential for establishing an accurate diagnosis of spontaneous TDIH and that the repair of a ruptured diaphragm with simple interrupted sutures without any prosthetic materials seems to be feasible in selected patients with a TDIH.

## Data Availability

Not applicable.

## Abbreviations

CT: Computed tomography
TDIH: Transdiaphragmatic intercostal hernia
